# CO_2_ Curing Efficiency for Cement Paste and Mortars Produced by a Low Water-to-Cement Ratio

**DOI:** 10.3390/ma13173883

**Published:** 2020-09-02

**Authors:** Seong Ho Han, Yubin Jun, Tae Yong Shin, Jae Hong Kim

**Affiliations:** Department of Civil and Environmental Engineering, Korea Advanced Institute of Science and Technology, Daejeon 34141, Korea; ha8890@kaist.ac.kr (S.H.H.); ssjun97@gmail.com (Y.J.); tyshin@kaist.ac.kr (T.Y.S.)

**Keywords:** CO_2_ curing, size effect, colloidal silica, cement-based material, casting method

## Abstract

Curing by CO_2_ is a way to utilize CO_2_ to reduce greenhouse gas emissions. Placing early-age cement paste in a CO_2_ chamber or pressure vessel accelerates its strength development. Cement carbonation is attributed to the quickened strength development, and CO_2_ uptake can be quantitatively evaluated by measuring CO_2_ gas pressure loss in the pressure vessel. A decrease in CO_2_ gas pressure is observed with all cement pastes and mortar samples regardless of the mix proportion and the casting method; one method involves compacting a low water-to-cement ratio mix, and the other method comprises a normal mix consolidated in a mold. The efficiency of the CO_2_ curing is superior when a 20% concentration of CO_2_ gas is supplied at a relative humidity of 75%. CO_2_ uptake in specimens with the same CO_2_ curing condition is different for each specimen size. As the specimen scale is larger, the depth of carbonation is smaller. Incorporating colloidal silica enhances the carbonation as well as the hydration of cement, which results in contributing to the increase in the 28-day strength.

## 1. Introduction

Greenhouse gas emissions in the industrial sector are of serious concern. In the construction industry, a large amount of CO_2_ is emitted during the production of cement by the calcination process. Various studies have been conducted to reduce the amount of CO_2_ emitted in the manufacturing process or to utilize emitted or captured CO_2_ for sustainable development [1,2]. CO_2_ curing for cement-based materials has been demonstrated as one possible way of utilizing CO_2_ [3,4,5,6,7].

The carbonation of calcium silicates such as C_3_S, *β*-C_2_S, and *γ*-C_2_S in Portland cement generally occurs more quickly than their hydration. Therefore, the CO_2_ curing of early-age concrete facilitates faster development of its strength [8]. The carbonation of anhydrous alite (C_3_S) and belite (C_2_S) is expressed as [9]:

(1) $$3C_{3}S+\left(3-x\right){\mathrm{CO}}_{2}+nH\to C_{x}{\mathrm{SH}}_{n}+\left(3-x\right){\mathrm{CaCO}}_{3}$$

and

(2) $$2\beta {-C}_{2}S+\left(2-x\right){\mathrm{CO}}_{2}+nH\to C_{x}{\mathrm{SH}}_{n}+\left(2-x\right){\mathrm{CaCO}}_{3}$$

where C_x_SH_n_ refers to the calcium silicate hydrates of (CaO)*_x_*(SiO_2_)(H_2_O)*_n_*, simply denoted by C-S-H. The carbonation products are the calcium silicate hydrates and calcium carbonate (CaCO_3_). In addition, the calcium hydroxide, a product by the calcium silicate hydration, is also carbonated:

(3) $$\mathrm{Ca}{\left(\mathrm{OH}\right)}_{2}+{\mathrm{CO}}_{2}\to {\mathrm{CaCO}}_{3}+H_{2}O$$

The calcium carbonate, produced at an early age, precipitates in pores of the cement paste. Consequently, cement-based materials obtain pore refinement, leading to enhanced durability and strength [10].

The degree of carbonation was usually estimated by the mass-curve or mass-gain method [11,12,13]. Equation (4) calculates CO_2_ uptake by measuring the increase in the mass of intact samples. The increase in the mass corresponds to the mass of reacted CO_2_. The mass-curve method [11] then evaluates

(4) $${\mathrm{CO}}_{2}\;\mathrm{uptake}\;\left(\%\right)=\frac{{\mathrm{Mass}\;\mathrm{of}\;\mathrm{specimen}\;\mathrm{subjected}\;\mathrm{to}\;\mathrm{CO}}_{2}\;}{\mathrm{Initial}\;\mathrm{mass}\;\mathrm{of}\;\mathrm{specimen}}\times 100$$

The mass-curve method needs caution when monitoring the increase in the mass of samples in a chamber. The water presented in a sample partially vaporizes due to the heat production from the carbonation, and excess water vapor condenses on the chamber wall. The mass-gain method compensates the water amount lost by vaporization to reduce experimental error [12,13,14]. Equation (5) then expresses a calculation of CO_2_ uptake:

(5) $${\mathrm{CO}}_{2}\;\mathrm{uptake}\;\left(\%\right)=\frac{\left(\mathrm{The}\;\mathrm{mass}\;\mathrm{increase}\;\mathrm{of}\;a\;\mathrm{cast}\;\mathrm{sample}\;\mathrm{by}\;{\mathrm{CO}}_{2}\;\mathrm{curing}\right)+\left(\mathrm{water}\;\mathrm{loss}\right)}{\mathrm{Mass}\;\mathrm{of}\;\mathrm{raw}\;\mathrm{materials}\;\mathrm{for}\;a\;\mathrm{sample}}\times 100$$

Another way to consider the CO_2_ uptake evaluation is by measuring the decrease in CO_2_ gas pressure in a sealed reactor. The decrease in the CO_2_ pressure monitored for the whole carbonation process can be converted into the CO_2_ mole consumed for the carbonation. Such a pressure monitoring method is less prone to error. This paper, therefore, proposes the pressure monitoring method.

The efficiency of CO_2_ curing is related to water in the pore system of a sample. Water invariably allows for the mixing and subsequent workability of cement-based materials. However, if there is much free water in the sample, water also fills the pore of a sample fully, resulting in hindering CO_2_ gas from entering the sample interior. CO_2_ diffusion into a sample is limited if its pores are fully saturated. On the other hand, CO_2_ in gaseous form does not react, and so its dissolution in liquid water precedes for the carbonation. Sufficient water, that is, more than the reacting amount accounted for in Equations (1) and (2), is required for the CO_2_ dissolution. Previous studies [15,16] suggested the use of a relatively low water-to-cement ratio (W/C less than 0.25) in CO_2_ curing, and made samples by compaction molding. The cement compacts produced by the low water-to-cement ratio had a large amount of air-filled pores, which resulted in a higher CO_2_ diffusion and CO_2_ uptake.

Controlling the pore system, by the use of nano-sized particles, also affects the efficiency of CO_2_ curing. The nucleation effect of nano-sized limestone powder on CO_2_ curing was reported [17,18]. In addition to the nano-sized limestone powder, this study further investigates the effect of incorporating colloidal silica. The nano-sized silica particles reportedly nucleate the hydration of cementitious materials in accompany with their pozzolanic reaction [19]. As a result, it reduces the degree of chloride ion penetration [20,21] even though the increase in compressive strength is not substantial [22]. Lastly, the optimization for CO_2_ curing conditions, together with the effect of specimen size, is also investigated for the purpose of controlling the pore system.

## 2. Experimental Details

### 2.1. Materials

Ordinary Type I Portland cement and ISO standard sand (ISO 679) [23] were used to produce samples in this study. Table 1 shows the chemical composition of the cement determined by X-ray fluorescence spectrometry. The specific density of the sand was 2620 kg/m^3^. Its grain size ranged from 0.08 to 1.60 mm. The colloidal silica (commercial grade) used in this study mainly contained particles from 10 to 20 nm, and the SiO_2_ content in the aqueous solution was 40%. The pH of the colloidal silica ranged from 9.5 to 10.5.

### 2.2. Sample Preparation

Table 2 lists the mix proportions of the samples. A planetary mixer was used for a total of 5 min mixing. The mixes were then fabricated by two methods: (1) Compacting or (2) conventional consolidating-in-mold procedure following ASTM C109 [24]. The compacting method was applied to the samples with a relatively low W/C: Paste (W/C = 0.15) and Mortar (W/C = 0.35), which considered the condition of brick production in practice. Each mix was filled in a confined mold, and then it was compacted by 5 kN compression for 30 min. The dimensions of the paste and mortar compacts were as a 40-mm cube. In contrast, the dimensions for the samples, Paste (W/C = 0.4) and Mortar (W/C = 0.5), produced by the conventional procedure [24], were various as 25-, 40-, and 50-mm cubes so as to analyze the specimen size effect on CO_2_ curing. The sealed curing for the premature sample in a mold proceeded for 24 h at approximately 25 °C.

An additional two mortar samples incorporating colloidal silica were produced to analyze the effect of colloidal silica. Their mix proportions and fabrication methods were the same as the samples in Table 2, with 4% colloidal silica per cement mass.

### 2.3. CO_2_ Curing and Successive Hydration

Table 3 details the schedule of the casting and curing conditions. We considered two conditions for the CO_2_ curing after demolding. The first CO_2_ curing condition was at 20% CO_2_ concentration, where the relative humidity (RH) was 75% ± 5% and the temperature was 25 °C under ambient pressure. Each sample was placed in a controlled chamber, and the 20% concentration CO_2_ curing continued for 28 days.

The other was 3 bar pressure CO_2_ curing. The samples in a pressure vessel, vacuum-sealed beforehand, were subjected to 99.9% purified CO_2_ gas for 3 h. The initial CO_2_ gas pressure was set above 340 kPa, but not to exceed 380 kPa (approximately 350 kPa). Each sample in Table 2 was subjected to 3 bar CO_2_ curing for 3 h, and then successive hydration followed: 21 h moisture curing for Paste (W/C = 0.15) and Mortar (W/C = 0.35), and 28 days water curing for Paste (W/C = 0.4) and Mortar (W/C = 0.5). The moisture curing was conducted under 85% ± 5% RH and a temperature of approximately 25 °C. The water curing was conducted at approximately 23 °C.

The control samples were produced by the moisture curing for the Paste (W/C = 0.15) and Mortar (W/C = 0.35). The control samples for Paste (W/C = 0.4) and Mortar (W/C = 0.5) took the water curing. The conditions for the controlled curing were the same as the conditions for the successive hydration after the 3 bar CO_2_ curing.

### 2.4. Measurements

**Figure 1:**
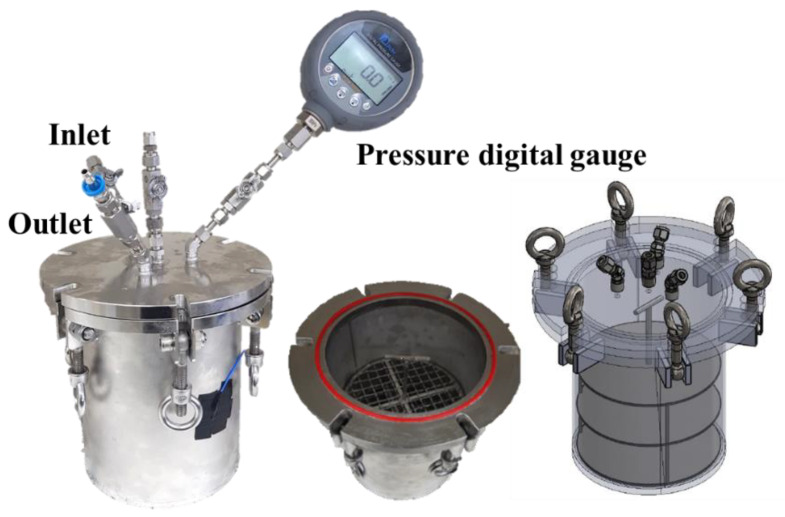
Pressure vessel.

Figure 1 shows a pressure vessel designed to have a single inlet valve and a single outlet valve. The internal temperature and pressure were monitored during the 3 bar CO_2_ curing. Pressure loss caused by cement carbonation was measured using a pressure digital gauge (PDR1000; Pressure Development of Korea Co., Daejeon, Korea). Its measurement range was from −100 kPa to 400 kPa, and its precision was 0.1%. The sampling rate for the pressure measurement was 1 record per second.

The compressive strengths of the samples were measured at the age of 1, 3, 7, 14, and 28 days in accordance with ASTM C109 [24]. The standard test method suggests a loading rate within 0.9 to 1.8 kN/s. In this study, the loading rate for the paste and mortar samples were assigned as 1.0 kN/s and 1.5 kN/s, respectively. The strength measurements took an average of the results of three replicated samples.

Each broken specimen following the strength measurement was used to evaluate the depth of carbonation. The depth of carbonation was determined using 1% phenolphthalein indicator in the broken specimen. The sprayed phenolphthalein solution on the specimen remains colorless when the specimen was carbonated, resulting in pH < 9.

## 3. Results

### 3.1. CO_2_ Uptake by Pressure Monitoring

**Figure 2:**
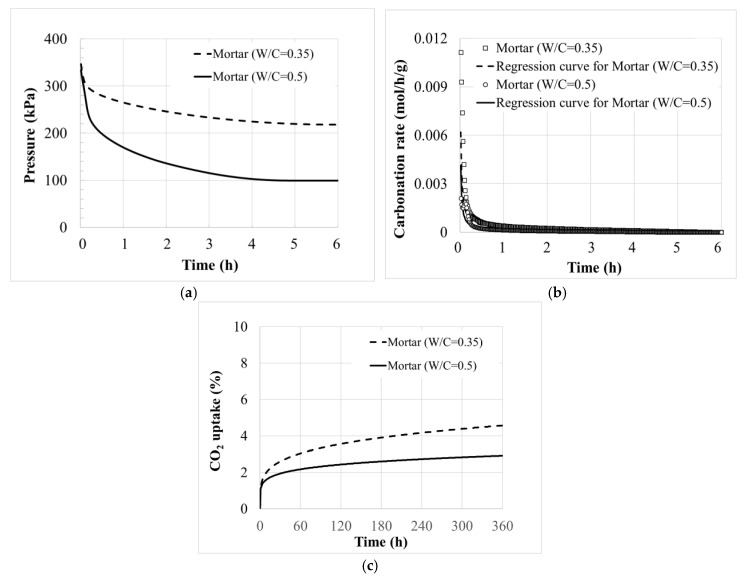
Pressure loss and CO_2_ uptake of Mortar (W/C = 0.35) and Mortar (W/C = 0.5). (**a**) Decrease of CO_2_ pressure, (**b**) Carbonation rate, (**c**) CO_2_ uptake.

Figure 2a shows the pressure loss of each pressure vessel during the 3 bar CO_2_ curing of Mortar (W/C = 0.35) and Mortar (W/C = 0.5) as an example. The values were calibrated by considering a CO_2_ leakage of 0.696 kPa/h in the pressure vessel. The CO_2_ pressure decreased over time, where the initial pressure was approximately 350 kPa as injected. Taking the slope at each point, the carbonation rate in a unit of kPa/h was evaluated as shown in Figure 2a. CO_2_ in the pressure vessel was also dissolved in the water phase of a sample, but the dissolution in water phase went to equilibrium quickly. The rate of the pressure decrease, disregarding the initial pressure records right after the initiation of the monitoring, therefore directly indicated the CO_2_ amount being carbonated.

The consumed CO_2_ was then calculated with the ideal gas equation, *PV* = *nRT*, where *R* is the gas constant of 8.314 J/mol/K. The volume of the pressure vessel was *V* = 0.004 m^3^. The temperature slightly changed over time, but it was averaged at *T* = 24 °C or 301 K. The consumed CO_2_, *n = PV/RT* per unit time, represented the carbonation rate in a unit of mol/h. Normalizing the carbonation rate with the cement mass required for producing the samples in the pressure vessel yielded its value per cement mass, as shown in Figure 2b. Integrating the carbonation rate for the time of the CO_2_ curing gave the CO_2_ uptake of each sample, where the percentage was calculated with the molecular weight of CO_2_ (44.01 g/mol) as shown in Figure 2c.

In Figure 2a, it can be seen that Mortar (W/C = 0.5) had a fast loss of pressure. However, as shown in Figure 2c, the actual amount of carbonation should be divided by the cement mass compared to the total mass. The result indicates that the CO_2_ uptake of the Mortar (W/C = 0.35) is larger than the CO_2_ uptake of Mortar (W/C = 0.5). As a result, it could be confirmed that the Mortar (W/C = 0.35) compact showed quicker carbonation, and with that, CO_2_ uptake, compared with the Mortar (W/C = 0.5) sample.

Table 4 compares the CO_2_ uptake per cement mass, where its value at 3 h is representatively reported. First of all, as expected, the Paste (W/C = 0.15) and Mortar (W/C = 0.35) compacts proportioned by a low W/C ratio had a higher CO_2_ uptake compared with the Paste (W/C = 0.4) and Mortar (W/C = 0.5) samples. The compacts made of low W/C had a high air-filled porosity, resulting in easy CO_2_ diffusion inside. Second, there was a size effect on the CO_2_ uptake of the paste samples. The 40-mm cube specimens showed a higher CO_2_ uptake than the 25-mm cubes of the replicated samples. Lastly, incorporating colloidal silica increased the CO_2_ uptake of the mortar samples. This will be discussed later in detail.

### 3.2. Compressive Strength

**Figure 3:**
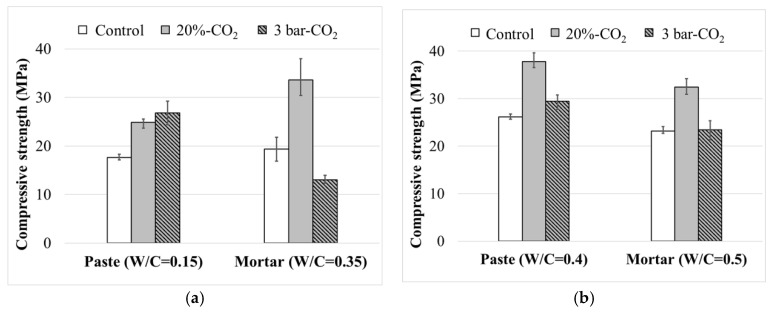
Strength of 1-day samples fabricated by (**a**) compacting and (**b**) consolidating-in-mold method.

Figure 3 compares the 1-day strengths of the 40-mm cube samples. The CO_2_ curing was influential in the early-age strength development of the paste samples. The strengths of Paste compact (W/C = 0.15) and Paste sample (W/C = 0.4) subjected to CO_2_ curing were higher than the control sample (moisture curing for 24 h). The 20% CO_2_ curing for 24 h was much more effective in the Paste samples (W/C = 0.4), while the 3 bar CO_2_ curing was better in the Paste compacts (W/C = 0.15). The effectiveness of the CO_2_ curing condition bifurcated with the mortar samples. The 20% CO_2_ curing resulted in a higher strength regardless of the casting method, whereas the 3 bar CO_2_ curing failed.

**Figure 4:**
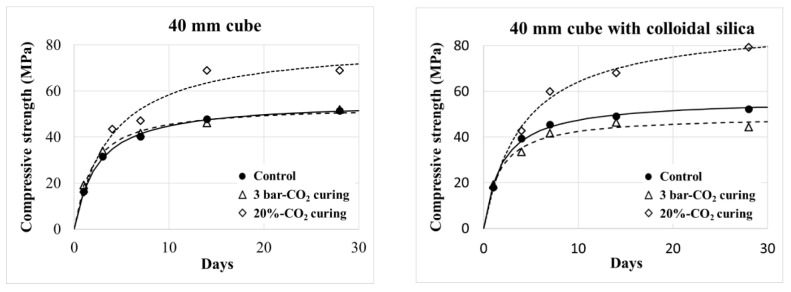
Strength development of Mortar (W/C = 0.5).

Figure 4 shows the strength development of the 40-mm cube samples of Mortar (W/C = 0.5), where each trend was fitted in a hyperbolic equation. Following the trend of the early-age strength, as illustrated in Figure 3, the 20% CO_2_ curing provided a higher strength gain than the other curing conditions. Incorporating colloidal silica intensified the effect of the 20% CO_2_ curing, which resulted in higher 28-day strength. However, the effect of the 3 bar CO_2_ curing was negligible, as shown in Figure 4, and even negative for Mortar (W/C = 0.5) incorporating colloidal silica.

## 4. Discussions

### 4.1. Optimization for CO_2_ Curing Condition

Both the initial pressure level and the duration in which the samples remained in the pressure vessel affect the efficiency of the CO_2_ curing. A high pressure of CO_2_ reportedly accelerates the carbonate reaction at an early age [25,26]. In this study, the initial pressure was therefore controlled for all samples: 3 bar, strictly inbetween 340 to 380 kPa, as stated in the previous section. For the effect of the duration, a preliminary test was conducted to optimize the CO_2_ curing condition using the pressure vessel. The duration of the CO_2_ curing then took the period in which the carbonation rate slowed to a crawl. As shown in Figure 2, the 50-mm cube Mortar (W/C = 0.35) and Mortar (W/C = 0.5) samples were cured for more than 6 h. The carbonation rate of Mortar (W/C = 0.35) is 1.71 × 10^−4^ mol/h/g at 3 h, and 0.98 × 10^−4^ mol/h/g at 6 h. The carbonation rate of Mortar (W/C = 0.5) is 5.40 × 10^−5^ mol/h/g at 3 h, and 3.02 × 10^−5^ mol/h/g at 6 h. The carbonation rate decreased from 3 h to 6 h was within 5% compared to the carbonation rate decreased during the initial 1 h. The carbonation under the initial pressure of 350 kPa was almost accomplished within 3 h.

The time of demolding is also critical for the effectiveness of CO_2_ curing on the sample produced by the conventional consolidating-in-mold procedure [27]. The time of sealed curing in a mold affects the air-filled pore system [16]. Cement hydration is expected to consume water and at the same time, also produce solid hydrates in the pores. The former increases the volume of air-filled pores, but the latter adversely decreases the total amount of pores. Another preliminary test was conducted for this case. The Paste (W/C = 0.4) and Mortar (W/C = 0.5) were sealed in 40-mm cube molds for 6, 12, 18, and 21 h, and then they were subjected to 3 bar CO_2_ curing after their demolding. Table 5 lists their CO_2_ uptakes. Note that the Mortar (W/C = 0.5) sample was broken when it was demolded at 6 h. Paste (W/C = 0.4) and Mortar (W/C = 0.5) had the highest CO_2_ uptake with the demolding time of 12 h and 18 h, respectively. After that, the CO_2_ uptake monotonically decreased with the demolding time. The air-filled pores were expected to decrease stably with the cement hydration. The time of 21 h for the demolding was therefore taken for the period when the air-filled pores showed stable change.

### 4.2. Effect of Specimen Size

**Figure 5:**
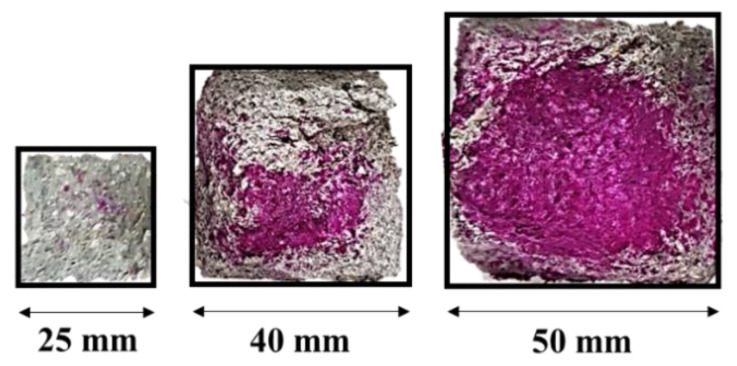
Carbonation depth of Mortar (W/C = 0.5) cured in 20% concentration CO_2_ for 28 days.

The size effect on the strength of cement-based materials is inherent, and CO_2_ curing affects the size effect of sample because of inconsistent CO_2_ diffusion. Figure 5 shows the carbonation depth of Mortar (W/C = 0.5) subjected to 20% CO_2_ curing for 28 days, where the area of carbonation can be clearly compared. The 25-mm cube specimen was fully carbonated, but its 40-mm and 50-mm cubes were not fully carbonated, displaying a crimson color inside (pH > 9).

The size effect law [28,29] helps us to understand the measurement of the compressive strengths of concrete. A large concrete cylinder provides a lower strength than that of a small cylinder which is geometrically similar to the large one. The tendency could be fitted with a size-effect equation [30]. Applying it to the current measurement generates an equation predicting the strength of a *D*-sized cube, *f_cu_*(*D*), based on that of a 25-mm cube:

(6) $$f_{cu}\left(D\right)=\frac{f_{cu}\left(25\right)}{{\left[1+\left(\frac{D}{\lambda_{0}d_{a}}\right)\right]}^{1/2}}B+\alpha f_{cu}\left(25\right)$$

where *α*, *B*, *λ*_0_, and *d_a_* can be considered empirical constants. Each parameter, notably *λ*_0_ and *d_a_*, has a physical meaning; however, here it is important that the parameters are constant. The variation of each strength is then explained by a linear relation of:

(7) $$\frac{\partial f_{cu}\left(D\right)}{\partial f_{cu}\left(25\right)}=\frac{1}{{\left[1+\left(\frac{D}{\lambda_{0}d_{a}}\right)\right]}^{1/2}}B+\alpha$$

**Figure 6:**
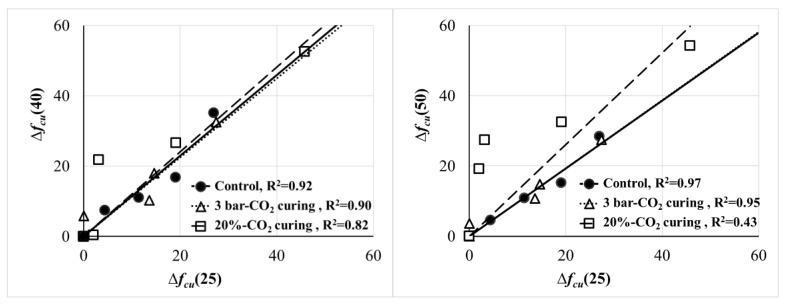
Comparison of the size effect on the strength development of mortar (W/C = 0.5).

Using Equation (7) allows us to consider the strength development of each sample. For example, Δ*f_cu_* (25) is calculated by the difference in the 25-mm cube strength at a certain age compared with 28 days (the reference age). Figure 6 comparatively shows the strength variation (development) of Mortar (W/C = 0.5). The linear trend lines, whose slopes correspond with the constant in Equation (6), do not change according to CO_2_ curing when the 25- and 40-mm cube strengths are compared: Δ*f_cu_*(25) and Δ*f_cu_*(40) in the left figure. However, the linear trend lines of the samples subjected to the 20% CO_2_ curing go off on the trend with the 50-mm cube strength (Δ*f_cu_*(25) and Δ*f_cu_*(50) in the right figure). The resultant nonlinear trend indicates that the size-effect parameters in Equation (6) need corrections or its reformulation. Partial carbonation on the edge of the 50-mm cube specimen, as shown in Figure 5, breaks the assumption of a geometrically similar specimen, which results in the nonlinear trend. In order to fit into the size effect law, the degree of carbonation in specimens with different size should be similar under the same CO_2_ curing condition. However, in this study, as the specimen size is larger, the depth of carbonation is smaller. This result may show that the size effect law of the specimen in CO_2_ curing does not fit.

### 4.3. Effect of Colloidal Silica on CO_2_ Curing

Colloidal silica reportedly contributes to a low diffusivity in a hardened cement paste and a low degree of carbonation because it has the filling/nucleating effect [20,31]. However, the CO_2_ curing produced an opposite effect. Incorporating the colloidal silica in the mortar samples subjected to the 3 bar CO_2_ curing increased the CO_2_ uptake approximately 56% for Mortar (W/C = 0.35) and 19% for Mortar (W/C = 0.5), as documented in Table 4. Incorporating non-reactive nanoparticles reportedly result in a slight increase in the rate of cement hydration because they provide supplementary nucleation sites [32,33,34]. Colloidal silica provides nucleation sites for the carbonation as well as the hydration of cement. Colloidal silica is more effective in the high-rate carbonation during which cement hydration is dormant. As a result, carbonation by 3 bar CO_2_ curing (at the age of 3 h) is accelerated.

Conversely, as shown in Figure 4, the strength of the mortar incorporating colloidal silica was not enhanced, but even weakened by the 3 bar CO_2_ curing. The carbonation products, mostly calcite, are not helpful for improving strength. Calcites are crystallized, and they do not provide a binding force among aggregates. Their inclusion in a paste matrix even cuts a binding link of the main hydrates (C–S–H). The strength of the mortar is consequently less developed.

When the samples incorporating colloidal silica were continuously subjected to 20% CO_2_ curing, their strengths increased up to 15% at 28 days. Colloidal silica certainly accelerates carbonation with 20% CO_2_ curing. Here, as opposed to the short-period 3 bar CO_2_ curing, carbonation continues concurrently with cement hydration in 20% CO_2_ curing. Calcite crystals produced by the carbonation put C–S–H on themselves at nano-scale, and the C–S–H layer is consequently reinforced by the distributed calcite [35,36,37]. The continuous 20% CO_2_ curing consequently improves the strength of the mortar sample.

## 5. Conclusions

Curing by CO_2_ can accelerate and improve the strength of cement-based materials via cement carbonation. In this study, 3 bar CO_2_ curing was applied to premature cement paste and mortar for 3 h, and then successive conventional curing followed for cement hydration. As a result, the strength of a paste compact (W/C = 0.15) increased a lot, providing a high CO_2_ uptake. That of a Paste (W/C = 0.4) consolidated in a mold also displayed a meaningful increase. However, despite cement carbonation, 3 bar CO_2_ curing resulted in an adverse effect in terms of the strength of a mortar compact (W/C = 0.35), while that of a mortar (W/C = 0.5) consolidated in a mold was unchanged. In contrast, continuous 20% CO_2_ curing increased the strengths of all the cement paste and mortar samples. Partial carbonation inside a specimen affects the size effect on the strength of the cement mortar. Incorporating colloidal silica provides more nucleation sites for cement carbonation, with the result that the effect of 20% CO_2_ curing is slightly improved.

## Figures and Tables

**Table 1:** Chemical composition of ordinary Portland cement (wt.%).

CaO	SiO_2_	Al_2_O_3_	Fe_2_O_3_	SO_3_	MgO	Na_2_O	K_2_O
67.29	17.18	4.13	4.17	3.16	1.94	0.22	1.23

**Table 2:** Mix proportion of samples.

Label	Fabrication Method	Mix Proportion (g)			
		Water	Cement	Sand	Colloidal Silica
Paste (W/C = 0.15)	Compacting ^†^	150	1000	0	–
Mortar (W/C = 0.35)		157.5	450	1350	–
Mortar (W/C = 0.35) with colloidal silica		157.5	450	1350	18
Paste (W/C = 0.4)	Consolidating-in-mold ^‡^	400	1000	0	–
Mortar (W/C = 0.5)		225	450	1350	–
Mortar (W/C = 0.5) with colloidal silica		225	450	1350	18

^†^ Each stiff sample in a confined mold was compressed by 5 kN (within 30 min). ^‡^ The mortar flow of each sample was within 150 to 250 mm.

**Table 3:** Curing condition and sequence for samples.

Label	Fabrication Time	Initial Curing for 24 h		Curing Until 28 Days
		3 h	21 h	
Paste (W/C = 0.15) Mortar (W/C = 0.35)	0.5 h	Moisture curing (Control)		
		3 bar CO_2_ curing	Moisture curing	
		20% concentration CO_2_ curing		
Paste (W/C = 0.4) Mortar (W/C = 0.5)	24 h	Water curing (Control)		
		3 bar CO_2_ curing	Water curing	
		20% concentration CO_2_ curing		

**Table 4:** Results of CO_2_ uptake.

Sample	CO_2_ Uptake at 3 h (%)	
	40-mm Cube	25-mm Cube
Paste compact (W/C = 0.15)	17.54	15.80
Paste sample (W/C = 0.4)	1.43	0.80
Mortar compact (W/C = 0.35)	9.51	–
Mortar sample (W/C = 0.5)	2.60	–
Mortar compact (W/C = 0.35) with colloidal silica	14.85	–
Mortar sample (W/C = 0.5) with colloidal silica	3.11	–

**Table 5:** CO_2_ uptake depending on sealed time.

Sample	Demolding Time (h)	CO_2_ Uptake (%)
Paste (W/C = 0.4)	6	0.76
	12	1.31
	18	0.89
	21	0.74
Mortar (W/C = 0.5)	6	–
	12	3.06
	18	3.77
	21	2.74
